# Hydrogen gas inhalation protects against cutaneous ischaemia/reperfusion injury in a mouse model of pressure ulcer

**DOI:** 10.1111/jcmm.13704

**Published:** 2018-06-19

**Authors:** Wei Fang, Guizhen Wang, Luyan Tang, Huilin Su, Huyan Chen, Wanqing Liao, Jinhua Xu

**Affiliations:** ^1^ Department of Dermatology Huashan Hospital Fudan University Shanghai China; ^2^ The Shanghai Institute of Dermatology Shanghai China; ^3^ Department of Dermatology and Venereology Changzheng Hospital Shanghai Key Laboratory of Molecular Medical Mycology Shanghai China; ^4^ Emergency room Shanghai Tenth People's Hospital of Tongji University Shanghai China

**Keywords:** hydrogen, inflammation, oxidative stress, pressure ulcer, reperfusion injury, wound healing

## Abstract

Pressure ulcer formation depends on various factors among which repetitive ischaemia/reperfusion(I/R) injury plays a vital role. Molecular hydrogen (H_2_) was reported to have protective effects on I/R injuries of various internal organs. In this study, we investigated the effects of H_2_ inhalation on pressure ulcer and the underlying mechanisms. H_2_ inhalation significantly reduced wound area, 8‐oxo‐dG level (oxidative DNA damage) and cell apoptosis rates in skin lesions. H_2_ remarkably decreased ROS accumulation and enhanced antioxidant enzymes activities by up‐regulating expression of Nrf2 and its downstream components in wound tissue and/or H_2_O_2_‐treated endothelia. Meanwhile, H_2_ inhibited the overexpression of *MCP‐1*,* E‐selectin*,* P‐selectin* and *ICAM‐1* in oxidant‐induced endothelia and reduced inflammatory cells infiltration and proinflammatory cytokines (TNF‐α, IL‐1, IL‐6 and IL‐8) production in the wound. Furthermore, H_2_ promoted the expression of pro‐healing factors (IL‐22, TGF‐β, VEGF and IGF1) and inhibited the production of MMP9 in wound tissue in parallel with acceleration of cutaneous collagen synthesis. Taken together, these data indicated that H_2_ inhalation suppressed the formation of pressure ulcer in a mouse model. Molecular hydrogen has potentials as a novel and alternative therapy for severe pressure ulcer. The therapeutic effects of molecular hydrogen might be related to its antioxidant, anti‐inflammatory, pro‐healing actions.

## 1 INTRODUCTION

Pressure ulcer is a chronic inflammatory dermatosis primarily occurring over bony prominences (such as the sacrum, trochanter and the heels) of senior bedridden patients. Without prompt treatment, pressure ulcer may progress to cellulitis, osteomyelitis, sepsis and even death. Pressure ulcer has become a serious public health problem owing to aging of the population.^1^

There are several hypotheses for the mechanisms underlying chronic pressure ulcer, such as local tissue hypoxia, repetitive ischaemia/reperfusion (I/R), wound bacterial colonization, etc.^2^, ^3^ Among these factors, I/R injury is thought to be a principal causative factor.^4^, ^5^ Long‐time recumbent position interrupts arteriolar capillary blood flow and thus induces local ischaemia in cutaneous tissue with prominent bony protrusions. After a change of body position, reperfusion of blood to the ischaemic skin initiates a series of harmful events because of a large increase in reactive oxygen species (ROS).^6^ Excessive ROS cause direct damage on lipids, proteins and nucleic acids, leading to cell apoptosis and tissue injury. Meanwhile, these free radicals induce the development of inflammatory responses such as endothelial dysfunction, neutrophil and macrophage infiltration, production of proinflammatory cytokines and, thereafter, tissues necrosis.^6^, ^7^ Consistently, several studies demonstrated that skin ulcers induced by cutaneous I/R were inhibited by treatment with antioxidants such as vitamin E, melatonin and deferoxamine.^8^, ^9^, ^10^ Therefore, antioxidant administration may be a good therapeutic strategy for promoting healing of pressure ulcers.

In the past decade, H_2_ as a novel medical gas has gained wide attention. In 2007, Ohsawa and colleagues found that H_2_ affords neuroprotection against brain I/R injury by selectively neutralizing hydroxyl radicals and peroxynitrite.^11^ Later studies demonstrated similar protective effects of hydrogen on I/R injuries of other organs such as liver, heart and intestines.^12^, ^13^, ^14^ The potential mechanisms might be involved in its antioxidant and anti‐inflammatory and anti‐apoptotic properties.^15^ These biological effects further enhanced the potential of H_2_ in clinical application of various organ system diseases.^15^, ^16^ Moreover, molecular hydrogen displayed high safety in vivo even at high concentration and high pressure, which could effectively reach target tissues and cells by gaseous diffusion but has no effect on physiological variables such as pH, oxygen saturation and blood pressure.^17^, ^18^ Taken together, we hypothesized that H_2_ could act as an effective treatment for pressure ulcer induced by repeated cutaneous I/R injury.

A recent study reported that hydrogen–water intake promoted wound size reduction and early recovery in 22 elderly in‐patients with severe pressure ulcer.^19^ However, there has been no experimental evidence of the beneficial effects of molecular hydrogen on I/R‐induced pressure ulcer using animal models. This study aimed to determine the possible protective effects of H_2_ on pressure ulcer and the underlying mechanisms.

## 2 MATERIALS AND METHODS

### 2.1 Animals and cells

Female C57BL/6 mice (8 to 12 weeks old) were purchased from Changzhou Cavens Laboratory Animal Ltd. (Changzhou, China). All experiments were approved by the Ethical Committee for Animal Experiments of Fudan University, and strictly carried out in accordance with the approved guidelines. HUVEC cells were purchased from ATCC (Manassan, VA), and maintained in RPMI 1640 containing 10% foetal bovine serum (Thermo Fisher Scientific Inc., Waltham, MA).

### 2.2 I/R cycles and analysis

The cutaneous I/R model was established according to previously published reports.^20^, ^21^, ^22^ Briefly, all mice were anaesthetized, and their backs were shaved and cleaned with 75% ethanol. The dorsal skin was gently pulled up and placed between two round ferrite magnetic plates that had a 12‐mm diameter and 5‐mm thickness (NeoMag Co, Ichikawa, Japan). A single I/R cycle was initiated with a 12‐hour period of magnet placement, and followed by a release or rest period of 12 hours. After three I/R cycles, all of the mice developed two circular ulcers separated by a bridge of normal skin. For analysis, each wound site was digitally photographed after wounding, and wound areas were measured on photographs using Image J (version 1.48, NIH, Bethesda, MD) as previously described.^20^, ^23^ To assess the effects of hydrogen gas on wound healing, the mice were housed in a specific airtight device producing air mixture including 2% or 75%H_2_ and 21%O_2_ for one week (6 hours per day) before the beginning of I/R cycles. H_2_ treatment was continuously performed until the wounds completely healed. The hydrogen‐producing device was provided by Shanghai Asclepius Meditec Co. Ltd (Shanghai, China). Wound sites were digitally photographed at various time‐points after wounding, and wound areas were measured on the images using ImageJ software version 1.46r (NIH, Bethesda, MD).

### 2.3 Histological and immunohistochemical examinations

The wounds were harvested with a 5‐mm rim of unwounded skin tissue from sacrificed mice. Skin samples were fixed in 10% paraformaldehyde and embedded in paraffin. Sections (6 μm) were stained with HE and Masson or processed for subsequent immunostaining. For immunohistochemistry, deparaffinized sections were incubated with 3% H_2_O_2_ for 5 minutes to block endogenous peroxidase activity. After blocking with 10% foetal bovine serum, sections were stained with primary antibodies of interest followed by secondary Abs. Sections were washed three times with PBS buffer. The colour was developed using DAB substrate‐chromogen solution (Biocare Medical). The sections were then counterstained with HE.

### 2.4 ROS measurement

ROS levels were measured in wound homogenates using ROS ELISA Kit (J&L Bio., Shanghai, China). For ROS detection in vitro, HUEVC cells were incubated in 2% or 75% H_2_ incubator (Shanghai Asclepius Meditec Co. Ltd) for 24 hours and then stimulated with 0.25 mmol/L H_2_O_2_ (100 mL/well) for 2 hours as previous report.^23^ The control group was without H_2_ pretreatment. The ROS levels were then examined with dihydroethidium (DHE) (Beyotime, Shanghai, China) according to the manufacturer’ protocol. Fluorescent pictures were collected by fluorescence microscope (Olympus IX71), and fluorescence intensity was analysed by Image J software (NIH, Bethesda, MD).

### 2.5 CCK8 assay

The rate of cell proliferation was detected using CCK8 assay kit (Beyotime, Shanghai, China). After H_2_ preincubation and 0.25 mmol/L H_2_O_2_ treatment, 10^4^ of HUEVC cells were placed into 96‐well plate (200 μL per well) and 20 μL CCK8 was added into each well for 24 hours. The optical density (OD) was read at 450 nm wavelength by ELISA microplate reader (Thermo Multiskan MK3).

### 2.6 Apoptosis (TUNEL) assay

Apoptosis assay was carried out in both skin sections and H_2_O_2_‐treated HUEVC cells using terminal deoxynucleotide transferase dUTP nick end labelling (TUNEL) staining kit (Roche Diagnostics, Indianapolis, IN) according to the manufacturer's instructions. Photographs were taken and visualized with inverted fluorescence microscope (Olympus IX71). The number of apoptotic cells was determined by counting TUNEL and Hoechst double positive nuclei in the field as previously described.^23^

### 2.7 Quantitative real‐time PCR

For real‐time PCR, total RNA was isolated from injured skin samples or H_2_O_2_‐treated HUVEC cells using the Rneasy kit (QIAGEN Ltd., Crawley, UK) according to the manufacturer's protocols. First‐strand cDNA was synthesized using the SuperScript III First‐Strand Synthesis Kit (Invitrogen) according to its protocol. Resulting cDNA was used as template for subsequent real‐time PCR using iQ SYBR Green Supermix (Bio‐Rad) according to the manufacturer's recommendations. Primers (Table S1) were synthesized by Shanghai Sangon Biotech Co., Ltd (Shanghai, China). Relative expression of PCR products was determined using the 2^−ΔΔCT^ method and calculated relative to the control group.

### 2.8 Western blot

For protein blotting, total proteins were extracted from homogenized skin tissue using as previously described.^24^ Twenty micrograms of proteins was loaded into 8% SDS‐PAGE and then transferred onto a polyvinylidene fluoride membrane. After blocking and washing, the membranes were incubated with the indicated primary Abs. The membranes were then incubated with horseradish peroxidase‐labelled secondary antibody and developed with the ImmobilonTW Western Chemiluminescent HRP Substrate (Millipore, Billerica, MA). The blots were assessed by Image J software (NIH, Bethesda, MD).

All the antibodies (Abs) and their sources in the study are listed in Table S2.

### 2.9 ELISA

Supernatants of mouse wound homogenates or cell culture medium were used for ELISA assay for SOD, GPx, CAT (BioVision), IL‐1β, TNF‐α, IL‐6, IL‐8 and IL‐22 (eBioscience) according to the manufacturers’ recommendation. Total protein in the supernatant was detected with a commercial kit (BCA Protein Assay kit; Pierce, Rockford, IL). The data were expressed as anti‐oxidative enzyme (unit/mL) or cytokine (pg/mL)/total protein (mg/mL) for each sample.

### 2.10 Statistical analysis

Results were expressed as means ± SEM. Unpaired, two‐sided Student's *t* test was performed determine the statistical differences between the sample means using GraphPad Prism 6.0 (La Jolla, CA). *P <* .05 were considered statistically significant.

## 3 RESULTS

### 3.1 H_2_ inhalation protected against cutaneous I/R‐induced pressure ulcer formation

**Figure 1:**
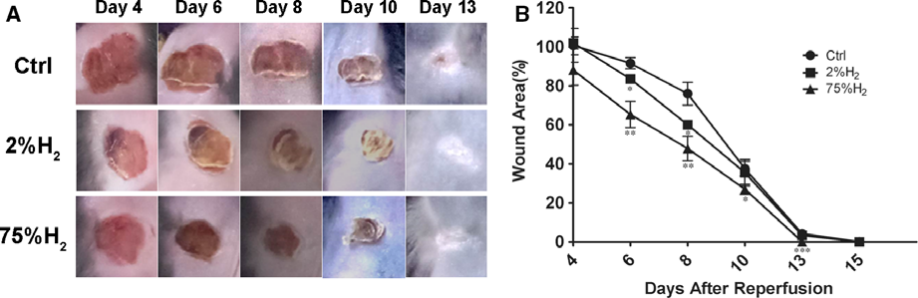
H_2_ inhalation protected against ulcer formation in cutaneous I/R injury mice model. A, Representative photographs of wounds after cutaneous I/R in control or H_2_‐treated mice at 4, 6, 8, 10 and 13 days after reperfusion. B, Relative wound area after I/R injury in normal C57BL/6 mice with or without H_2_ inhalation (N = 8 for each time point and groups). The ulcer size in control mice at 4 days after reperfusion was assigned a value of 100%. *P < .05; **P < .01; ***P < .001 compared to control

To assess the preventive effects of H_2_ on the development of cutaneous pressure ulcers after I/R injury in vivo, a decubitus ulcer‐like mouse model was constructed as previously described.^20^, ^23^ In order to reach fully saturation in cutaneous tissue, 2% or 75% H_2_ was inhaled by mice in experimental groups for one week (6 hours per day) before the beginning of I/R cycles according to the discovery of Scottish physiologist John Scott Haldane about the human body and the nature of gases.^25^ Wound areas in H_2_‐treated mice were significantly smaller than those in control mice especially in early stage after I/R cycles (Figure 1). Inhalation of 75% H_2_ significantly shortened the wound closure time in pressure ulcer mice. Furthermore, we also observed cleaner wounds and less scratching behaviour in H_2_‐treated mice than in control group. These results suggested that H_2_ inhalation especially at high concentration protected the formation of cutaneous ulcers after I/R cycles.

### 3.2 H_2_ inhalation altered the histopathological characteristics of pressure ulcer skin after cutaneous I/R

**Figure 2:**
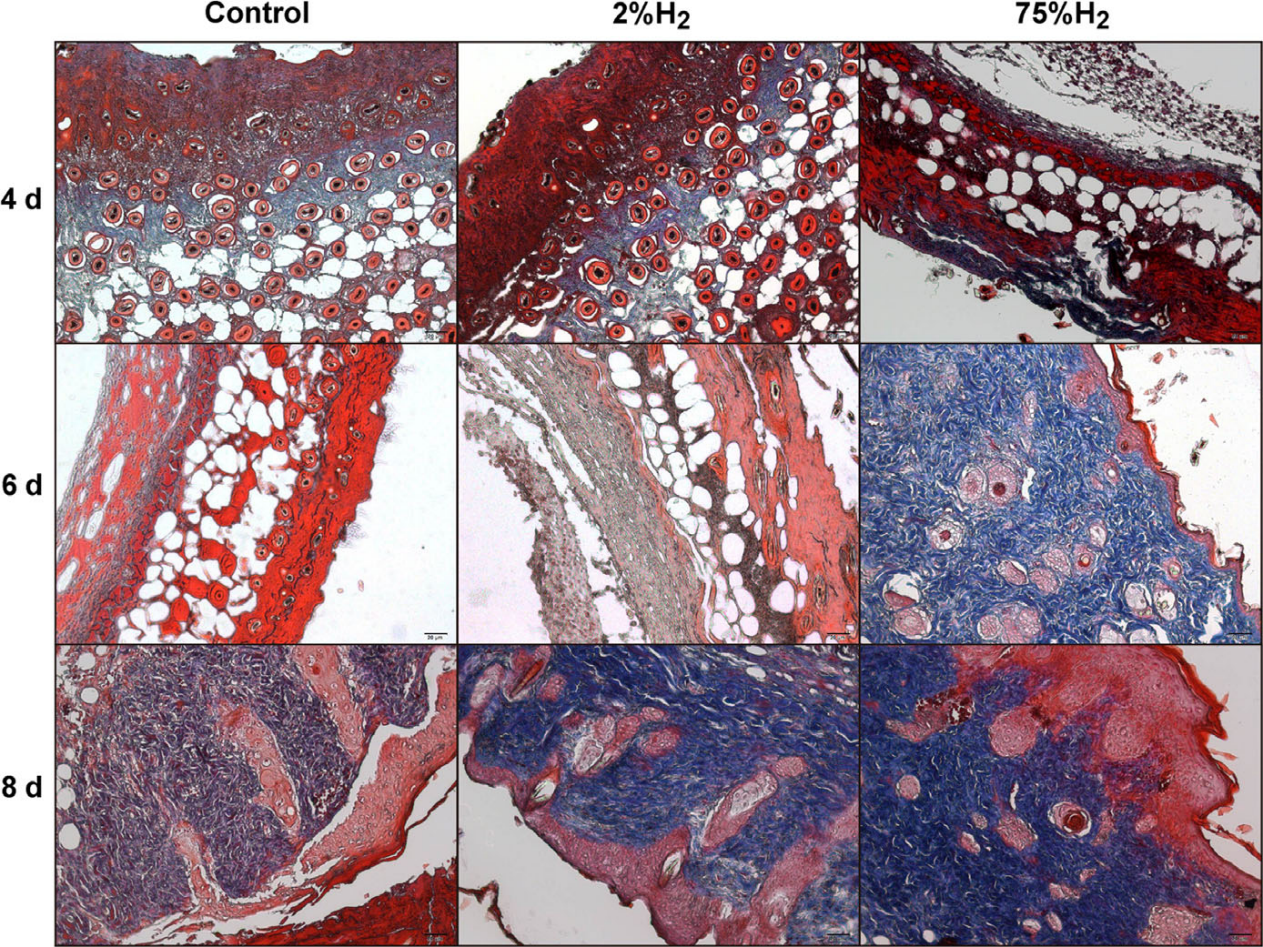
H_2_ treatment promoted dermal collagen synthesis in skin wound caused by I/R cycles. Skin tissue samples from pressure ulcer mice of each group were fixed and paraffin embedded. The tissue sections were subjected to Masson staining. Representative images from the wound skin tissues are shown. Scale bar, 20 μm

Haematoxylin and eosin (HE) staining showed that H_2_ inhalation reduced inflammatory cell infiltration and tissue necrosis in skin wound caused by I/R cycles (Figure S1). Compared with the control group, H_2_‐treatment groups (especially 75%H_2_) displayed notable acceleration and enhancement in dermal collagen synthesis of skin wound (Figure 2). These results suggested that H_2_ inhalation alleviated the inflammatory response and promoted the wound healing in pressure ulcer.

### 3.3 H_2_ inhalation alleviated oxidative DNA damage and suppressed apoptosis in skin tissues after cutaneous I/R

We further evaluated the levels of oxidative DNA damage (8‐oxo‐dG) and cell apoptosis after cutaneous I/R injury. Immunohistochemical (IHC) analysis showed that three groups had similar levels of 8‐oxo‐dG staining at 4 days postreperfusion. However, the area and intensity of positive staining in H_2_‐treated mice were significantly reduced compared to those in control mice and 75% H_2_‐treated group had lowest level of 8‐oxo‐dG at 6 and 8 days after reperfusion (Figure S2A). TUNEL analysis showed that wound tissues of control mice had a large number of apoptotic cells, which was significantly reduced by H_2_ inhalation (especially 75% H_2_) at day 8 postreperfusion (Figure S2B and Figure 2C). These results suggested that H_2_ inhalation could alleviate the oxidative DNA damage and suppress apoptosis of skin tissues, and high‐concentration H_2_ had optimal protective effect against cutaneous I/R injury.

### 3.4 H_2_ inhalation reduced ROS accumulation and up‐regulated antioxidant enzyme activities in skin tissue after cutaneous I/R

**Figure 3:**
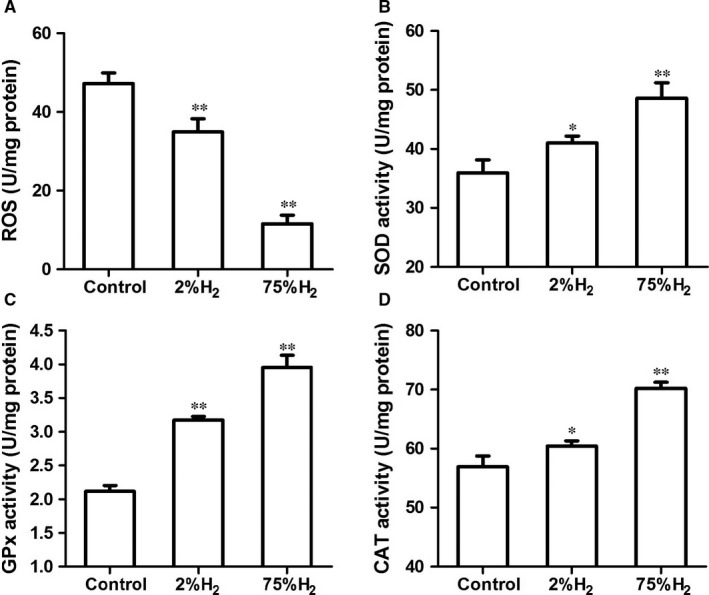
H_2_ inhalation reduced ROS accumulation and enhanced antioxidant enzyme activities in skin tissue. ELISA assays for detection of ROS production (A) and the activities of SOD (B), GPx (C), and CAT (D). *P < .05; **P < .01 compared to control

As ROS is essential mediators of reperfusion induced tissue damage,^26^, ^27^, ^28^, ^29^ we assessed ROS levels and the activities of major antioxidant enzymes including superoxide dismutase (SOD), catalase (CAT) and glutathione peroxidase (GPx) in the wound tissue at day 6 postreperfusion. H_2_ suppressed the ROS accumulation in skin tissue caused by I/R injury (*P *< .01) in a concentration‐dependent manner (Figure 3A). Activities of SOD, CAT and GPx in mice inhaled H_2_ were significantly higher than in control mice (Figure 3B‐D).

### 3.5 H_2_ activated the NRF2‐ARE pathway in skin tissue after cutaneous I/R

**Figure 4:**
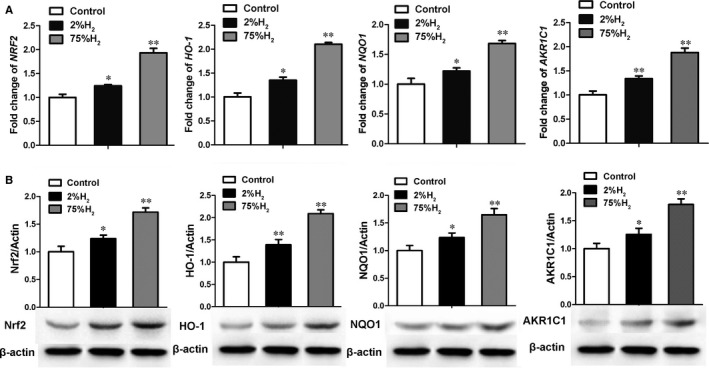
Hydrogen activated the NRF2‐ARE pathway in wounded tissue. A, The mRNA levels of NRF2, HO‐1, NQO1 and AKR1C1 were analysed by quantitative RT‐PCR assay. B, Immunoblots of NRF2, HO‐1, NQO1, AKR1C1 and β‐actin. *P < .05; **P < .01 compared to control

We next investigated whether NRF2/ARE antioxidant pathway mediated the inhibition of ROS level of pressure ulcer tissue by H_2_. Quantitative real‐time PCR analysis showed that H_2_ significantly up‐regulated the mRNA levels of *NRF2* and its target genes (heme oxygenase‐1, *HO‐1*; NADH quinone oxidoreductase 1, *NQO1*; aldo‐keto reductase, *AKR1C1*) in a concentration‐dependent manner in the skin tissue (Figure 4A). Immunoblotting also confirmed the enhanced expression of NRF2‐ARE pathway components in the skin tissue of H_2_ treatment group (especially 75%) (Figure 4B). These results indicated that H_2_ activated the NRF2 pathway in skin lesions after cutaneous I/R.

### 3.6 H_2_ inhalation inhibited cutaneous I/R‐induced inflammation

**Figure 5:**
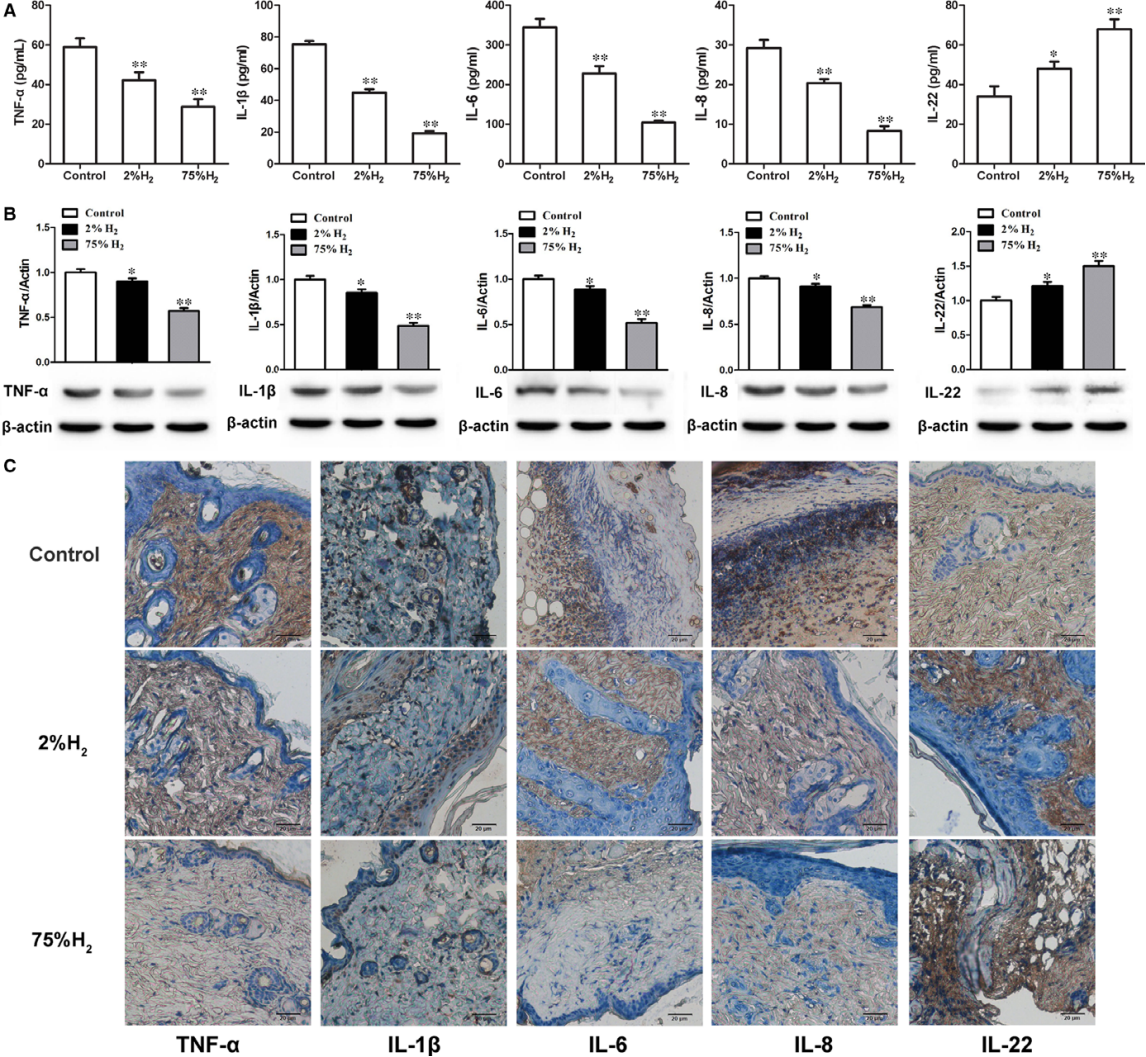
Hydrogen altered the expression or production of inflammatory cytokines during IR cycles. A, mRNA expression of TNF‐α, IL‐1β, IL‐6, IL‐8 and IL‐22 in skin wounds. B, Immunoblot detecting the protein levels of TNF‐α, IL‐1β, IL‐6, IL‐8 and IL‐22 in wounded skin tissue. C, IHC assessing injured skin TNF‐α, IL‐1β, IL‐6, IL‐8 and IL‐22 levels. *P < .05; **P < .01 compared to control

As proinflammatory cytokines are important for tissue damage during I/R injury, the levels of proinflammatory cytokines in the wounded skin were assessed. At day 6 post‐I/R, H_2_ treatment significantly reduced the relative mRNA and protein expression of TNF‐α, IL‐1β, IL‐6 and IL‐8 in wounded tissue, and their expression was lowest in pressure ulcer mice treated with 75% H_2_ (Figure 5A and B). IHC further confirmed that the protein levels of TNF‐α, IL‐1β, IL‐6 and IL‐8 in cutaneous I/R injured skin tissue of mice inhaled H2 were substantially lower than those of control mice (Figure 5C). Meanwhile, H_2_ treatment significantly up‐regulated the expression of IL‐22, an essential pro‐healing cytokine involved in repair events on different models of epithelial regeneration,^30^, ^31^ at both mRNA and protein levels and 75% H_2_ treated group displayed the highest level of IL‐22 (Figure 5), which was mainly expressed in the dermal layer of perilesional skin tissues in mice treated by H_2_ (Figure 5C). These results indicated that hydrogen inhalation reversed proinflammatory effects of skin wound environment after cutaneous I/R.

### 3.7 H_2_ inhalation modulated the expression of healing‐associated molecules in vivo after cutaneous I/R

**Figure 6:**
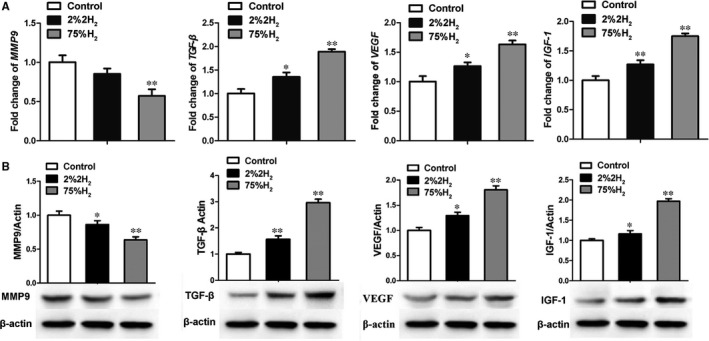
Hydrogen modulated the expression of healing‐associated molecules in skin wounds. A, mRNA expression of MMP9, TGF‐β, VEGF and IGF‐1 was analysed by quantitative RT‐PCR. B, Immunoblot assessment of MMP9, TGF‐β, VEGF and IGF‐1 protein levels in wounded skin tissue. *P < .05; **P < .01 compared to control

The metalloprotease MMP9 and pro‐healing factors TGF‐β, VEGF and IGF‐1 play crucial roles during proliferative and remodelling phases of wound healing. To investigate how H_2_ improves wound healing in pressure ulcer mice, its effects on expression of aforementioned molecules were analysed by quantitative PCR and immunoblotting. H_2_ inhalation significantly decreased the expression level of MMP9 and enhanced the expression of TGF‐β1, VEGF and IGF‐1 at both mRNA and protein levels in the wound. Mice treated with 75% H_2_ had the lowest level of MMP9 and highest expression of pro‐healing factors in skin tissue among three groups (Figure 6).

### 3.8 H_2_ reversed the adverse effect of H_2_O_2_ on cell proliferation and apoptosis of vascular endothelia cells

I/R injury is closely associated with microvascular dysfunction, which is largely a consequence of endothelia cell impairment.^7^ Thus, we further examine the effects of H_2_ on human umbilical vein endothelial cells (HUVECs) against oxidative damage. Preincubation with H_2_ for 24 hours significantly relieved the 0.25 mmo/L H_2_O_2_‐induced proliferation inhibition of HUVECs in a concentration‐dependent manner (*P *< .01) (Figure S3A). After exposure to 0.25 mmol/L H_2_O_2_, HUVECs showed a 2.5‐folds increase in the apoptosis rate, which was markedly reduced by H_2_ pretreatment (especially 75%H_2_) (*P *< .01) (Figure S3B and C).

### 3.9 H_2_ rectified the imbalance of oxidation‐reduction system in H_2_O_2_‐treated vascular endothelia cells through the activation of Nrf2/ARE pathway

To further understand the mechanism of H_2_‐mediated protective effects on endothelial cells during cutaneous I/R, a series of in vitro studies were performed in oxidant‐treated HUVECs. H_2_O_2_ notably up‐regulated intracellular ROS production (Figure S4A) and suppressed the expression of various anti‐oxidative enzymes (SOD, GPx, and CAT) (Figure S4B‐D) in HUVECs, suggesting of a severe disturbance of intracellular oxidation‐reduction system. Preincubation with H_2_ resulted in a trend towards normalization of both intracellular ROS and anti‐oxidative enzyme in H_2_O_2_‐treated HUVECs (Figure S4A‐D). Quantitative PCR assay demonstrated that H_2_ significantly up‐regulated the expression of *NRF2* and its target genes *HO‐1*,* AKR1C1* and *NQO1* in HUVECs exposed to H_2_O_2_ in a concentration‐dependent manner (Figure S4E‐H). Together, these results suggested that H_2_ might maintain intracellular homoeostasis of oxidation‐reduction system in vascular endothelial cells through the activation of Nrf2/ARE pathway.

### 3.10 H_2_ inhibited the expression of several chemokine and adhesion molecules in vascular endothelial cells stimulated by H_2_O_2_

**Figure 7:**
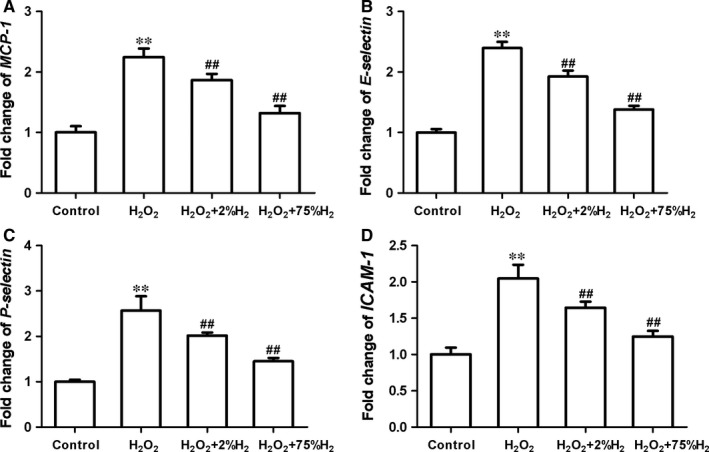
Hydrogen decreased the expression of several molecules associated with leucocyte‐endothelium in H_2_O_2_‐treated HUEVCs. Quantitative RT‐PCR assay of MCP‐1 (A), P‐selectin (B), E‐selectin (C) and ICAM‐1(D) in HUEVCs. **P < .01, *P < .05, compared to the control group; ^##^ *P *< .01, ^#^ *P *< .05, compared to H_2_O_2_‐treated group

Endothelial damage is a critical event in the early phase of inflammatory responses induced by cutaneous I/R, which could drive the up‐regulation of various chemokines and adhesion molecules and then mediate leucocyte recruit, adhesion and emigration.^7^, ^32^ Vascular endothelial cells more than doubled the transcriptional levels of *MCP‐1*(monocyte chemoattractant protein‐1), *E‐selectin*,* P‐selectin* and *ICAM‐1*(intercellular cell adhesion molecule‐1) after H_2_O_2_ exposure (Figure 7). Pretreatment with H_2_ significantly suppressed the elevation of their expression in H_2_O_2_‐exposed HUEVCs (*P *< .01) (Figure 7). The inhibitory effects were positively correlated with H_2_ concentration. Our results suggested that H_2_ might exert anti‐inflammatory effects against cutaneous I/R injury by down‐regulating the expression of endothelial chemokines and adhesion molecules.

## 4 DISCUSSION

In the present study, we first demonstrated the protective effects of H_2_ inhalation on pressure ulcers in a murine model. H_2_ treatment significantly reduced the wound area in a concentration‐dependent manner in the early phase after cutaneous I/R. The improvement in skin ulcer recovery induced by H_2_ was paralleled by significant reductions in oxidative DNA damage, cell apoptosis and acceleration of cutaneous collagen synthesis. This result was consistent with a clinical study that hydrogen–water intake (0.8‐1.3 ppm, 600 mL per day) via tube‐feeding significantly reduced the wound size in 22 hospitalized patients with pressure ulcer.^19^ Theoretically, hydrogen administration through respiratory route displays higher blood concentration and more accessibility for cutaneous lesions than those through gastrointestinal absorption.^15^ Therefore, H_2_ inhalation has a greater potential to be applied in the prevention and treatment of patients with pressure ulcer.

H_2_‐mediated protection against pressure ulcers was primarily dependent on its antioxidant property. Oxidative stress plays crucial roles in the initiation and progression phases of cutaneous I/R injury.^3^, ^6^, ^26^ In pressure ulcers, blood reperfution after cutaneous ischaemia induced abundant reactive oxygen species (ROS), which could directly damage skin tissue and cause cell apoptosis. These toxic free radicals further aggravated leucocyte infiltration, which in turn triggered more ROS release and thus trapped in a vicious circle. In our study, H_2_ treatment remarkably decreased the ROS accumulation and 8‐oxo‐dG formation (a sensitive biomarker of oxidative DNA damage), and also enhanced the activities of several antioxidant enzymes (such as SOD, GPx and CAT) in wound tissue and/or H_2_O_2_‐treated HUEVCs. The antioxidant ability was positively correlated with its dosage, and high concentration of H_2_ was more advantageous in maintaining intracellular homoeostasis of oxidation‐reduction system in skin tissue after I/R injury.

We also determined the mechanism underlying the antioxidant property of H_2_ against cutaneous I/R injury, which was closely associated with the activation of Nrf2/ARE pathway. The transcription factor Nrf2 is essential for regulating the adaptive response to exogenous and endogenous oxidative stresses.^33^, ^34^ Under moderate oxidative stress, Nrf2 translocates to the nucleus where it binds to ARE and induces the transcription of downstream antioxidant genes. In our study, H_2_ treatment significantly up‐regulated the expression of Nrf2 and its downstream targets such as HO‐1, NQO1 and AKR1C1 in wound tissue and HUEVCs exposed to H_2_O_2_. Similar phenomena were also observed in previous studies of other organ I/R injury or other inflammatory diseases.^35^, ^36^, ^37^, ^38^ Molecular hydrogen could attenuate intestinal injury in wild‐type but not NRF2‐knockout mice with severe sepsis by regulating HO‐1 expression.^37^ Therefore, hydrogen might activate the Nrf2/ARE pathway to restore the homoeostasis of cutaneous oxidation‐reduction system against I/R injury.

The protective effects of H_2_ were also attributed to its potent anti‐inflammatory activity. After I/R cycles, plenty of macrophages and neutrophils were accumulated in the treated tissue accompanied by significant production of proinflammatory cytokines, which caused skin injury and necrosis in the wound.^21^ In our study, H_2_ inhalation attenuated inflammatory cells infiltration and cutaneous necrosis in the wound induced by I/R injury. Proinflammatory cytokines such as TNF‐α, IL‐1, IL‐6 and IL‐8 were decreased while IL‐22 was increased in the wounded skin of H_2_‐treated mice. Distinct from proinflammatory cytokines, IL‐22 mediates a crosstalk between immune system and cutaneous cells (such as fibroblasts and keratinocytes) and plays a pro‐healing role in wound repairment.^30^, ^39^ Our data demonstrated that H_2_ reversed the excessive inflammatory response induced by cutaneous I/R injury.

The anti‐inflammatory property of hydrogen might be partially attributed to its protection on vascular endothelial cells against oxidative stress. During I/R injury, endothelial damage initiated leucocyte adhesion, recruitment and infiltration by up‐regulating the expression of chemokines and adhesion molecules.^6^, ^7^, ^32^ Our in vitro assay showed that H_2_ treatment significantly alleviated the oxidative injury of endothelial cells and reduced the overexpression of *MCP‐1*,* E‐selectin*,* P‐selectin* and *ICAM‐1* in HUEVCs exposed to H_2_O_2_. MCP‐1 is a critical molecule for chemotaxis and activation of macrophage, which is a significant source of proinflammatory cytokines and contributes to I/R injury of skin and other organs.^21^, ^40^, ^41^, ^42^ The adhesion molecules studied here were mainly responsible for leucocyte rolling, localization and adhesion to the endothelium.^43^, ^44^ Hence, hydrogen might modulate those molecules associated with leucocyte–endothelium interaction to inhibit subsequent inflammatory reaction caused by cutaneous I/R injury.

In pressure ulcer, the wound sites were constantly in the dynamic pathological alterations of inflammatory injury and tissue repair. Outcome of wound healing process was mainly determined by the presence and concentration of the healing‐associated factors such as MMPs, TGF‐β, VEGF and IGF1.^45^, ^46^, ^47^ Overproduction or high activity of MMP9 and suppressed expression of TGF‐β were identified as indicators of poor healing in skin samples of chronic ulcers.^46^, ^48^ In our study, H_2_ promoted the expression of pro‐healing factors (TGF‐β, VEGF and IGF1) and inhibited the production of MMP9 in wound tissue of pressure ulcer, accompanied by acceleration of dermal collagen synthesis. Hence, hydrogen had a wound healing promoting effect against cutaneous I/R injury.

Taken together, the present results indicated that hydrogen suppressed the formation of decubitus ulcers by its antioxidant, anti‐inflammatory, pro‐healing activities against cutaneous I/R injury. Similar therapeutic effects of hydrogen were also reported in common senile diseases concomitant with decubitus, such as cerebral or myocardial infarctions, COPD, diabetes, hyperlipaemia, malignant tumours.^11^, ^36^, ^49^, ^50^, ^51^, ^52^, ^53^, ^54^ Furthermore, H_2_ had no cytotoxicity *in vivo* in human body even at a high concentration.^17^ Therefore, hydrogen gas has a great potential for preventing and/or treating pressure ulcer.

There were still some limitations in our study. Firstly, we did not detect the cutaneous hydrogen concentration, which might provide direct evidence to support the dosage‐dependent protection of hydrogen gas against cutaneous I/R injury. In addition, the exact mechanisms by which hydrogen modulates oxidative stress, inflammation and wound repair in pressure ulcer were still unclear. More experiments are needed to work out these problems before the clinical trial of H_2_ in pressure ulcers.

## CONFLICT OF INTEREST

The authors have no conflicts of interest to report.
