# Supplementary figures and images for: Hydrogen gas inhalation protects against cutaneous ischaemia/reperfusion injury in a mouse model of pressure ulcer
Source: J Cell Mol Med. 2018 Jun 19;22(9):4243–52. doi: 10.1111/jcmm.13704 (PMC6111801; doi:10.1111/jcmm.13704)

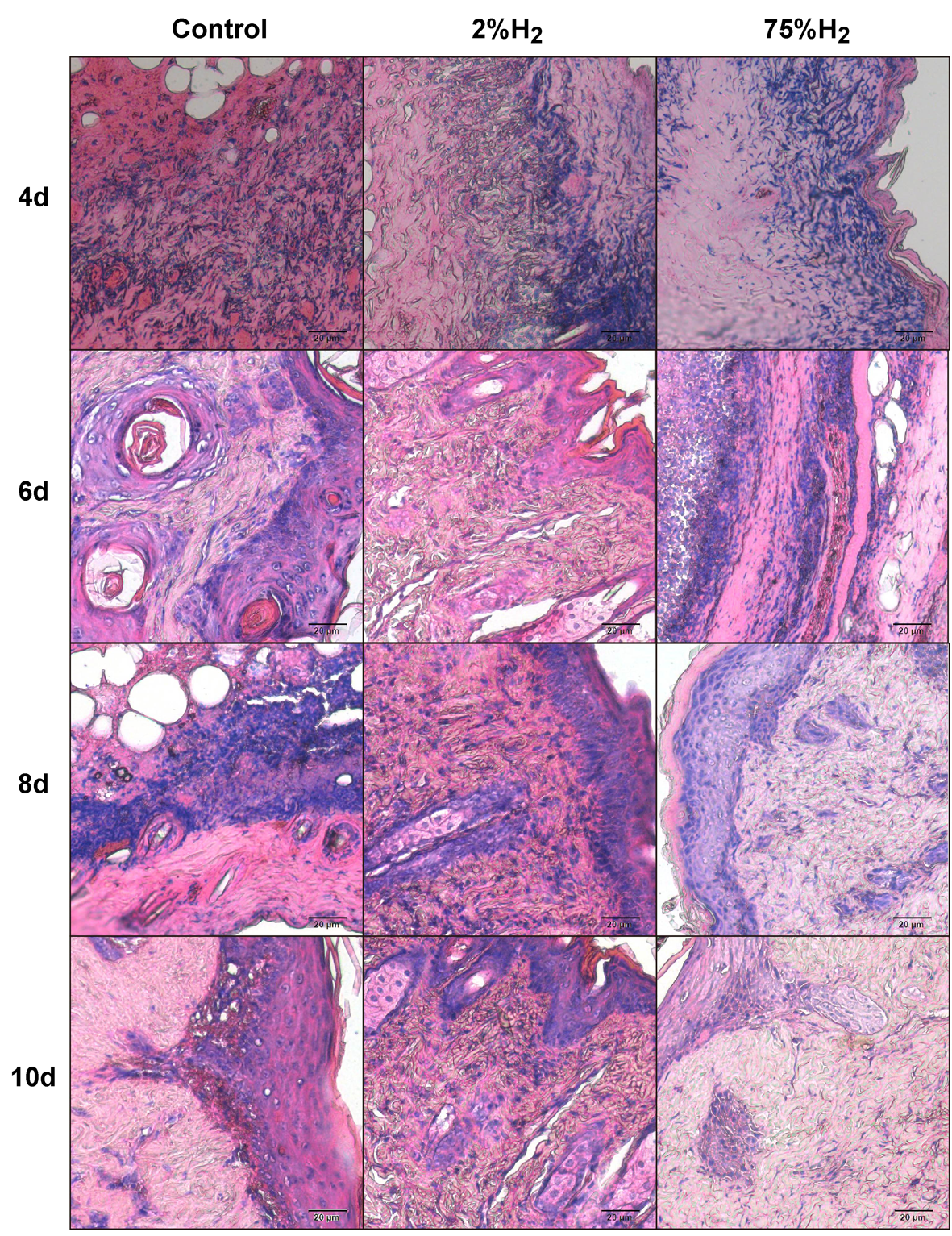

Supplement: Supplementary file 1 [file JCMM-22-4243-s001.tif]

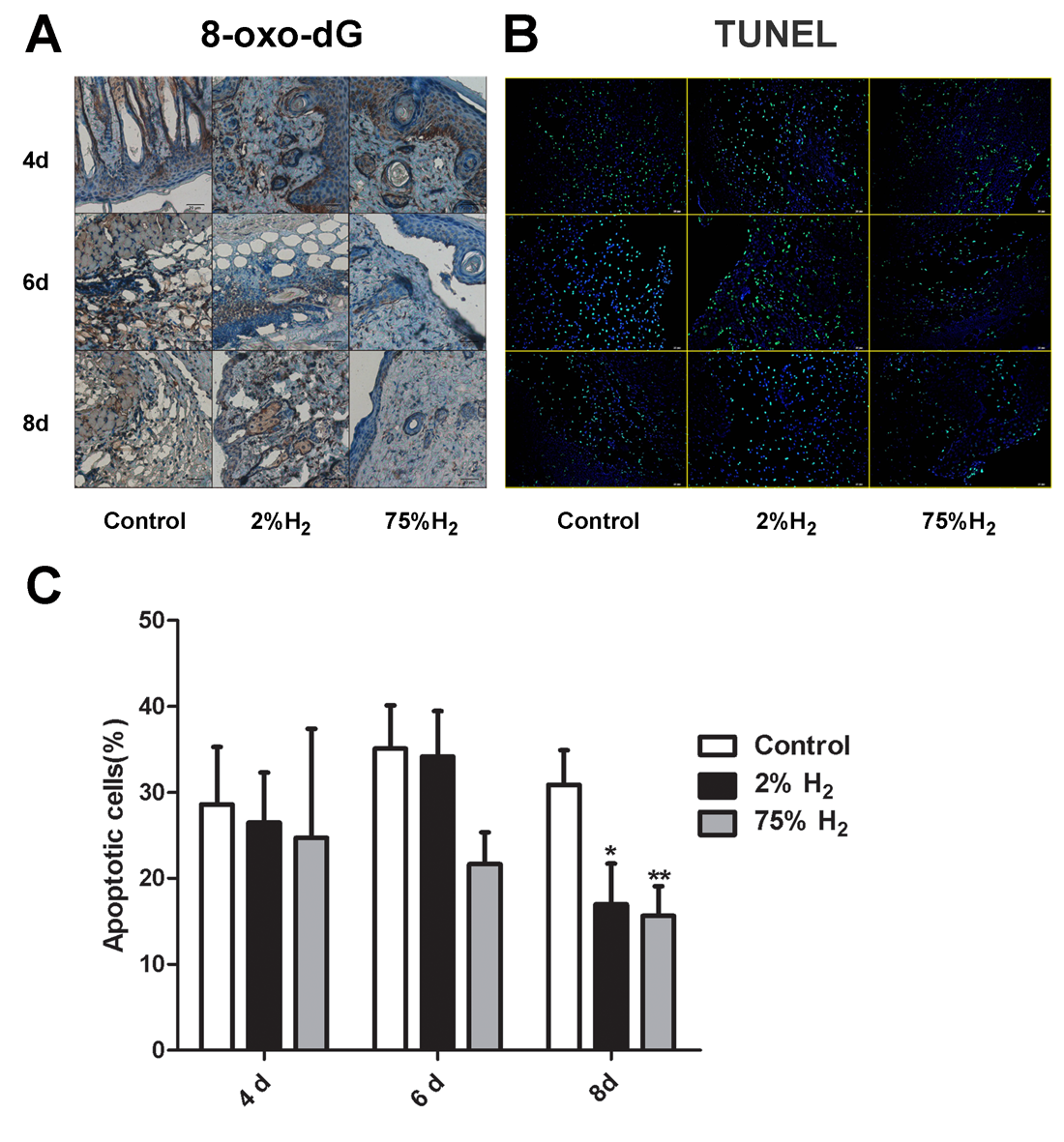

Supplement: Supplementary file 2 [file JCMM-22-4243-s002.tif]

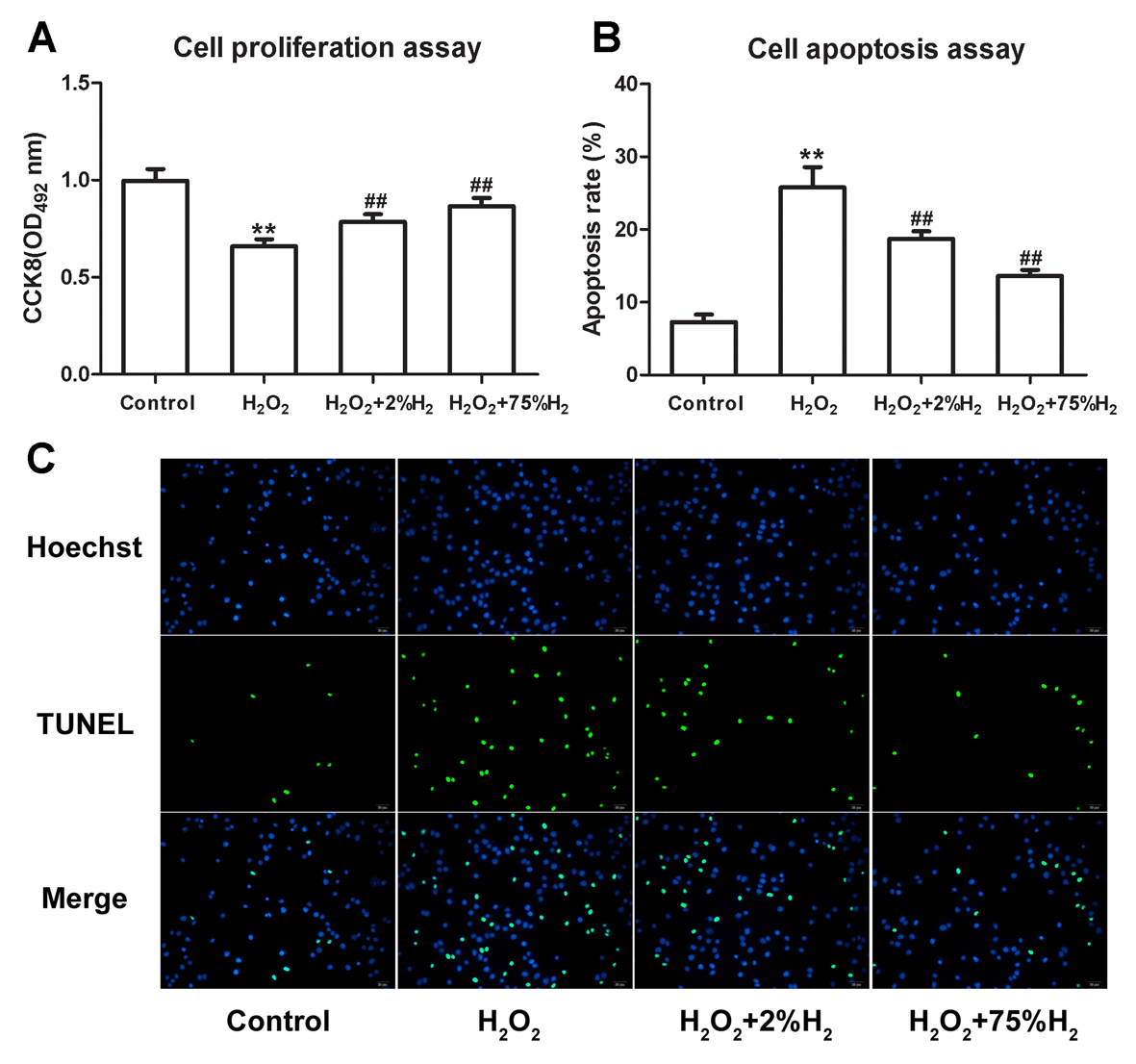

Supplement: Supplementary file 3 [file JCMM-22-4243-s003.tif]

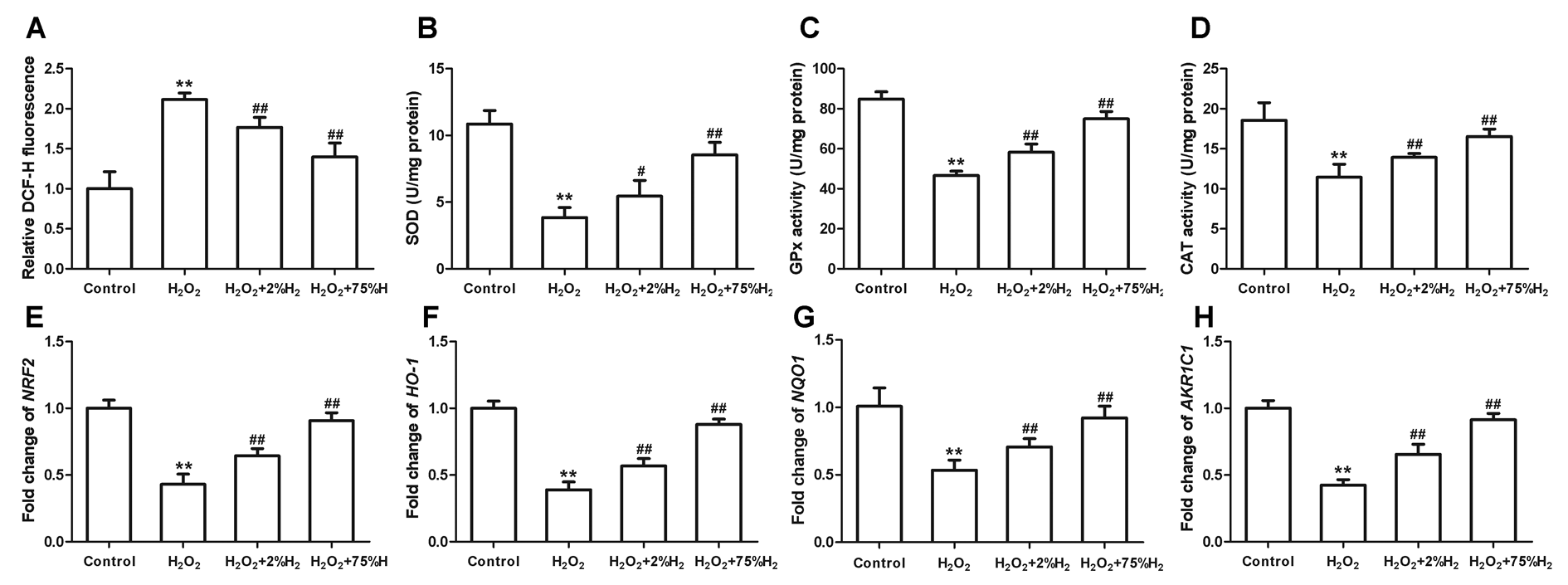

Supplement: Supplementary file 4 [file JCMM-22-4243-s004.tif]
