# Supplementary material for: Hydrogen gas inhalation protects against cutaneous ischaemia/reperfusion injury in a mouse model of pressure ulcer
Source: J Cell Mol Med. 2018 Jun 19;22(9):4243–52. doi: 10.1111/jcmm.13704 (PMC6111801; doi:10.1111/jcmm.13704)
Supplement: Supplementary file 5 [file JCMM-22-4243-s005.doc]

**Table S1**. Primers used in this study.

| **NO.** | **Primer** | **Sequence(5’-3’)** |
| --- | --- | --- |
| 1 | VEGF(mouse)-F | AGGCAGACTATTCAGCG |
| 2 | VEGF(mouse)-R | AGTGATTTGAGGGAGTG |
| 3 | TGF-β(mouse)-F | GGCGGTGCTCGCTTTGTA |
| 4 | TGF-β(mouse)-R | TTTCTCATAGATGGCGTTGTT |
| 5 | IGF-1(mouse)-F | GGTGGATGCTCTTCAGTT |
| 6 | IGF-1(mouse)-R | TTTGTAGGCTTCAGTGGG |
| 7 | MMP9(mouse)-F | TGGGACCATCATAACATCAC |
| 8 | MMP9(mouse)-R | ATGACAATGTCCGCTTCG |
| 9 | NRF2(mouse)-F | AGTGCTCCTATGCGTGAA |
| 10 | NRF2(mouse)-R | GCGGCTTGAATGTTTGTC |
| 11 | HO-1(mouse)-F | ACAGATGGCGTCACTTCG |
| 12 | HO-1(mouse)-R | TGAGGACCCACTGGAGGA |
| 13 | AKR1C1(mouse)-F | TGGTCACTTCATCCCTAT |
| 14 | AKR1C1(mouse)-R | GTGCCATCTGCTATCTTG |
| 15 | NQO1(mouse)-F | CTCTTCAGGGTGTCCACG |
| 16 | NQO1(mouse)-R | TCCTCCCAGACGGTTTCC |
| 17 | β-actin(mouse)-F | GTCCCTCACCCTCCCAAAAG |
| 18 | β-actin(mouse)-R | GCTGCCTCAACACCTCAACCC |

**Table S2. Antibodie**s used in this study.

| **NO.** | **Antibodies(Ab)** | **Sources** |
| --- | --- | --- |
| 1 | Rat anti-mouse 8-OHdG monoclonal Ab | (Santa Cruz Biotechnology, Santa Cruz, CA) |
| 2 | Rabbit anti-mouse Nrf2 monoclonal Ab | (Abcam, Cambridge, UK) |
| 3 | Rat anti-mouse NQO1 monoclonal Ab | (Santa Cruz Biotechnology, Santa Cruz, CA) |
| 4 | Rabbit anti-mouse HO-1 monoclonal Ab | (Abcam, Cambridge, UK) |
| 5 | Rabbit anti-mouse AKR1C1 monoclonal Ab | (Abcam, Cambridge, UK) |
| 6 | Rat anti-mouse TNF-α monoclonal Ab | (Abcam, Cambridge, UK) |
| 7 | Rat anti-mouse IL-1β monoclonal Ab | (Cell Signaling Technology, Danvers, MA) |
| 8 | Rabbit anti-mouse IL-6 monoclonal Ab | (Proteintech Group, Rosemont, IL) |
| 9 | Rabbit anti-mouse IL-8 monoclonal Ab | (Abcam, Cambridge, UK) |
| 10 | Rabbit anti-mouse IL-22 monoclonal Ab | (Abcam, Cambridge, UK) |
| 11 | Rabbit anti-mouse MMP9 monoclonal Ab | (Abcam, Cambridge, UK) |
| 12 | Rat anti-mouse TGF-β monoclonal Ab | (Abcam, Cambridge, UK) |
| 13 | Rat anti-mouse VEGF monoclonal Ab | (Abcam, Cambridge, UK) |
| 14 | Rabbit anti-mouse IGF-1 monoclonal Ab | (Abcam, Cambridge, UK) |
| 15 | Rat anti-mouse β-actin monoclonal Ab | (Proteintech Group, Rosemont, IL) |
| 16 | Goat anti-rabbit secondary Ab | (ZSGB-BIO, Beijing, China) |
| 17 | Goat anti-rat secondary Ab | (ZSGB-BIO, Beijing, China) |
